# Investigating the Effects of Polyaluminum Chloride on the Properties of Ordinary Portland Cement

**DOI:** 10.3390/ma12203290

**Published:** 2019-10-10

**Authors:** Taewan Kim, Choonghyun Kang, Sungnam Hong, Ki-Young Seo

**Affiliations:** 1 Department of Civil Engineering, Pusan National University, Busan 46241, Korea; ring2014@naver.com; 2 Department of Ocean Civil Engineering, Gyeongsang National University, Gyeongsangnam-do 53064, Korea; snhong@gnu.ac.kr; 3 HK Engineering and Consultants, Busan 46220, Korea; aricari@hanmail.net

**Keywords:** polyaluminum chloride, ordinary portland cement, Friedel’s salt, chloride ion

## Abstract

This study investigates the mechanical and microstructural properties of paste comprising ordinary Portland cement (OPC) added with polyaluminum chloride (PACl). The properties of the resulting mixture are analyzed using compressive strength, X-ray diffraction, scanning electron microscopy (SEM), mercury intrusion porosimetry, and thermogravimetric analysis. The results show that the addition of PACl improves the mechanical properties of OPC paste, that calcium-(aluminum)-silicate-hydrate (C-(A)-S-H) gel and Friedel’s salt are the major products forming from the reaction with the aluminum and chloride ions in PACl, and that the portlandite content decreases. Moreover, the size and number of micropores decrease, and compressive strength increases. All these phenomena are amplified by increasing PACl content. SEM images confirm these findings by revealing Friedel’s salt in the micropores. Thus, this work confirms that adding PACl to OPC results in a mixture with superior mechanical and microstructural properties.

## 1. Introduction

Polyaluminum chloride (PACl) is a coagulation agent that has recently begun to be used in water-treatment processes. It is more effective than the existing aluminum sulfate system, and its use is expanding [1,2,3]. Polyaluminum chloride is used in the water-treatment industry to coagulate organic and mineral colloids prior to sedimentation and/or filtration. The aluminum destabilizes fine colloidal suspensions and promotes the agglomeration of this material bound in a chemical precipitate (called floc), which can be removed from the water by sedimentation, flotation, and/or filtration. In general, PACl is preferred over aluminum sulfate if larger, faster-forming floc is desired, because it may remove the need for a flocculant to facilitate sedimentation [1,4,5].

PACl is applicable over a wide pH range [1,2,3]. It changes into various Al-hydroxide group depending on pH. PACl is hydrolyzed in water to form Al13-type polymers, which are typically referred to as stable forms of Keggin-A13 [2,6]. Keggin-Al13 adsorbs and aggregates the particles to form flocs [2]. PACl contains chloride in combination with various types of polymeric Al-groups. Therefore, ordinary Portland cement (OPC) mixed with PACl is expected to be changed by aluminum and chloride. In the early stages of hydration, the effect of aluminum on the OPC particle surface and the reaction of hydration reactants with chloride will affect the mechanical and microstructural properties. 

Previous studies reported that Friedel’s salt was observed in OPC exposed to chloride-containing environments [7,8,9,10]. Friedel’s salt is said to improve the chloride ion immobilization effect by mixing cementitious materials rich in aluminum ions [11,12,13,14,15]. Aluminum and chloride ions contained in PACl create an environment that can simultaneously affect OPC particles. Therefore, the effect of using PACl, which is used as a coagulation agent, as an admixture for OPC will be examined. The use of PACl in cement is rare. Chen et al. [16] experimented to mix PACl in powder form with a binder consisting of 60% OPC + 40% slag with 1% and 2% contents. As a result, PACl had the effect of improving strength and reducing voids. Kim [17] also published a study using 0–10% of liquid PACl in slag-based alkali-activated cement. The use of PACl in alkali activated cement (AAC) results in Friedel’s salt formation improving compressive strength, reducing pore diameter and total porosity, and calcium-(aluminum)-silicate-hydrate (C-(A)-S-H) gel formed. In the study with PACl, no study was applied to OPC. Therefore, we want to examine the effect of PACl on OPC. It is believed that this will provide an extensible basis for binders that combine OPC and various supplementary cementitious materials.

This research is an experimental study of the effect of PACl on the paste with OPC as the main binder. PACl was intended to be homogeneously distributed in paste using liquid type. The addition of PACl creates an environment in which chloride and aluminum ions exist simultaneously in the initial stage of OPC hydration. Therefore, this research aims to investigate the effect of PACl on the hydration reaction and mechanical properties of OPC. The effects of mechanical and microstructural properties were also investigated by performing compressive strength, X-ray diffraction (XRD), scanning electron microscopy (SEM), mercury intrusion porosimetry (MIP) and thermal analysis (thermogravimetric (TG)/differential thermal analysis (DTG)). The chloride ion contained in PACl should be used with caution because it causes corrosion of the rebar. If the experimental results of this study confirm the improvement of the mechanical performance of OPC by PACl, further studies will be conducted on the corrosion and durability of rebar. Subsequent research will be a research step on the properties of reinforced concrete mixed with PACl.This experiment is the first step to investigate the effect of PACl on OPC.

## 2. Materials and Methods 

### 2.1. Materials

Table 1 lists the components and physical properties of the ordinary Portland cement (OPC) used in these experiments. X-ray fluorescence (XRF, SHIMADZU XRF-1800, Tokyo, Japan) analysis was used to acquire these data. Table 2 lists the components and physical properties of polyaluminum chloride (PACl, liquid type). The PACl is a commercial product for treating drinking water. PACl is dosed as a liquid solution.

### 2.2. Experiments Methods

The water-binder ratio of the paste was 0.45. PACl added 0%, 2%, 4%, 6%, 8%, and 10% of the mass of the mixing water, and was combined with the mixing water before mixing with OPC. This study used liquid-phase PACl to add a given mass of mixing water because of the rapid agglomeration obtained when such a solution is mixed with a powdered binder.

The paste was mixed by the method of ASTM C305 [18]. PACl was premixed in mixing water. The mixed paste is placed in a 50 × 50 × 50 mm cube mold for compressive strength measurement. Then, by moist curing method, a temperature of 23 ± 2 °C and a relative humidity of 90% ± 5% are stored in a constant chamber for 24 h. After 24 h, the mold is removed and the paste samples are stored in a constant temperature and humidity chamber (23 ± 2 °C, 90% ± 5%) until the measurement date. The compressive strengths were measured at 3, 7, and 28 day ages. The average of three samples’ measurements was used. After measuring the compressive strength, the broken sample pieces are immersed in acetone for 24 h to stop hydration. It was then dried in a vacuum desiccator for 24 h to remove acetone, ground to a fine powder and subjected to X-ray diffractometer (XRD, PANalytical Empyrean, Almelo, Netherlands) analysis. XRD analysis was performed by Empyrean of PANalytical. The XRD analysis conditions are from 5° to 60° (2θ range), 40 mA, 45 kV, Cu-Kα radiation (λ = 1.54443 Å), and step size of 0.017º (2θ). XRD analysis was performed on 3 day and 28 day samples. 

The 28 day sample was measured by mercury intrusion porosimetry (MIP, micromeritics AutoPore IV9500, Norcross, GA, USA) for pore structure analysis. The MIP was cut into pieces at the center of the cube sample, immersed in 24 h acetone, and dried in a 24 h vacuum desiccator. The MIP was analyzed for pores with diameters ranging from 0.003 to 337 μm. The analysis conditions are contact angle 130°, surface tension 485 dyn/cm and mercury (Hg) density 13.534 g/mL. 

Scanning electron microscopy (SEM, Zeiss SUPRA.40, Oberkochen, Germany) for microstructural analysis was performed using energy dispersive spectroscopy (EDS, Elite, AMETEK, Mahwah, NJ, USA). The SEM for the 28 day sample was measured immediately after compressive strength measurement and after shredded pieces were dried in a 24 h vacuum desiccator after stopping 24 h hydration. Measurements were made using a high vacuum mode using an accelerating voltage of 15 kV.

Thermal analysis (thermogravimetric (TG)/ differential thermal analysis (DTG), DSC800, Perkin Elmer, MA, USA) was performed for 3 day and 28 day samples. The analysis conditions are in a temperature range of 30 °C to 850 °C at 20 °C/min in an N_2_-gas environment.

## 3. Results and Discussion

### 3.1. Hydration Products

**Figure 1:**
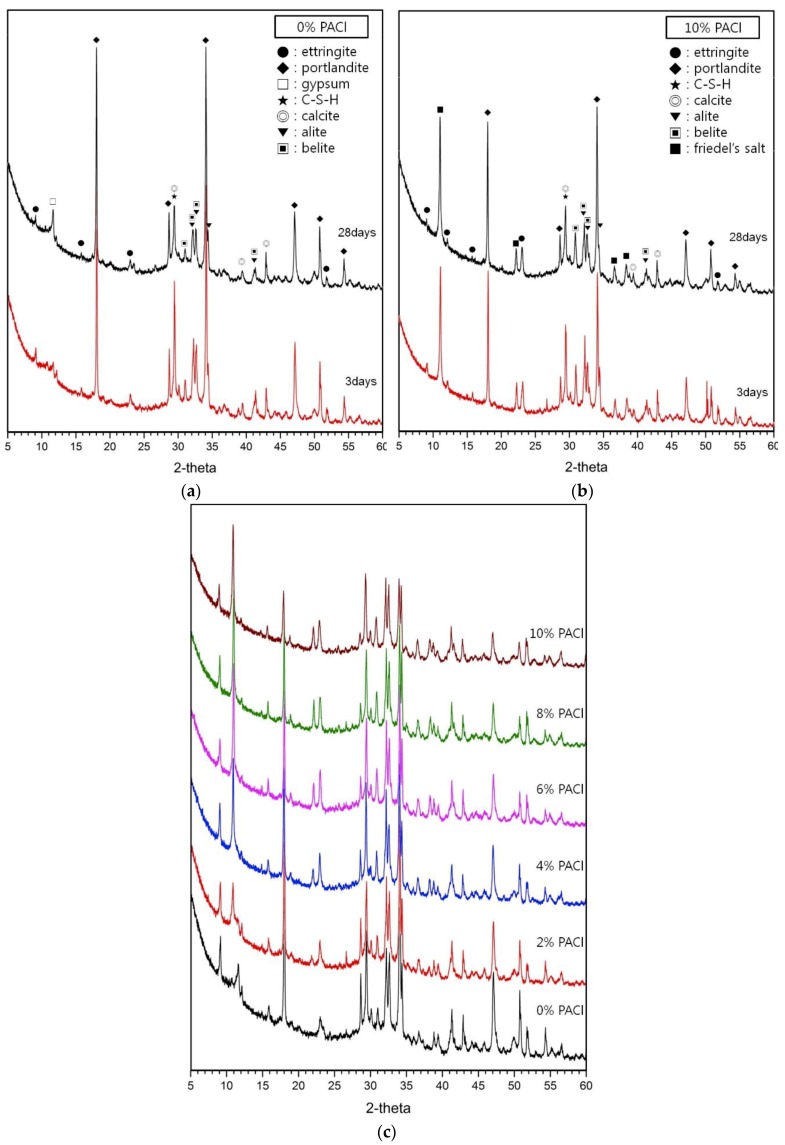
X-ray diffraction (XRD) analysis: (**a**) 0% PACl samples at 3 and 28 days, (**b**) 10% PACl samples at 3 and 28 days, (**c**) comparison of XRD spectra at 28 days.

Figure 1 shows the results of XRD analysis for the 0% and 10% PACl samples. The 0% PACl sample of Figure 1a contains ettringite, portlandite, calcite, belite, and alite. At 28 days, the portlandite peak is larger than at 3 days, whereas the alite and belite peaks are slightly smaller. However, the ettringite and calcium-silicate-hydrate (C-S-H) gel peaks maintain a relatively constant magnitude. Figure 1b shows the results for a 10% PACl sample. The main reaction products are Friedel’s salt, calcium-(aluminum)-silicate-hydrate (C-(A)-S-H) gel, and ettringite. The portlandite peak decreases with aging from 3 to 28 days, and the ettringite and C-(A)-S-H gel peaks decrease slightly. Compared with 0% PACl, the ettringite and C-(A)-S-H peaks are small, and the portlandite peak decreases sharply. In addition, Friedel’s salt appears in the 10% PACl sample.

The change in the height of the XRD peak of the reaction product is confirmed by comparing the 28 day hydration products with the increase of the PACl content, which is obtained from the data shown in Figure 1c. As the PACl content increases, the portlandite peaks clearly decrease and the peaks for Friedel’s salt clearly increase. In addition, the peaks for ettringite and C-(A)-S-H decrease significantly.

Friedel’s salts (3CaO·Al_2_O_3_·CaCl_2_·10H_2_O) are produced by the reaction between chloride ions and OPC hydration reactants [7]. Theories are being developed that describe clear mechanisms for the production and characterization of Friedel’s salt [6,7,8,9]. One mechanism provides two ways for chlorides to form Friedel’s salts: ion exchange (chemical binding) and absorption (physical binding) [10,11,12,13]. C-S-H gel is a typical candidate for chloride absorption [14,15]. Although it is less effective than C-S-H gel, ettringite can also bind chloride ions [10,11,19].

Previous studies have examined several factors affecting chloride-ion binding by C-S-H gel. The factors that are known to affect binding are type and composition of supplementary cementitious materials [7,20], and the properties of C-S-H (Ca/Si and Ca/Al ratios) [14]. Other studies have shown that portlandite also adsorbs chloride ions, although the results for portlandite are different than for C-S-H gel. Elakneswaran et al. [21] showed that chloride ions may be adsorbed on the surface of portlandite, but this suggestion was contested by Hirao et al. [22]. Therefore, the most we can say at this moment is that the capacity of portlandite and ettringite to adsorb chloride ions is relatively low compared with that of C-S-H gel. In a study by Shi et al. [23], researchers report that calcium ions play an important role in chloride binding. Therefore, it can be assumed that the decrease of portlandite is consumed by the role of chloride ion absorption or Friedel’s salt change.

In the present study, the magnitude of the portlandite XRD peak decreases upon adding PACl, whereas the ettringite or C-(A)-S-H peak do not increase. Despite the increase in age and PACl content, chloride binding is considered to cause the small increase in the XRD peaks of C-(A)-S-H and ettringite. The variability of C-(A)-S-H and ettringite with the concentration of PACl is not clear by XRD analysis. Therefore, we will refer to it later in the thermal analysis.

The PACl aluminum ion reacts with C-S-H gel to form the denser C-(A)-S-H gel as the main hydration product of OPC [16,21,22]. C-S-H is the main hydration product in Portland cements and contributes significantly to its physical, chemical, and mechanical properties. The C-S-H phase is poorly crystalline (“gel-like”) with variable calcium, silica, and water content. C-S-H can be described as a calcium-oxide layer sandwiched by silica chains organized in a “dreierketten” structure, which is a repeating chain of three silica tetrahedra [24]. Two of these silica units, called “pairing tetrahedral units,” are linked to the calcium-oxide layer, whereas the third unit (i.e., the bridging tetrahedron) links the two pairing tetrahedra. Water, calcium, alkalis or other ions are present in the interlayer between the layers [25]. Therefore, when the aluminum-ion concentration increases in the mixture due to addition of PACl, C-S-H gel adsorbs aluminum at the interlayer and changes to the C-(A)-S-H gel structure. Previous studies of PACl and OPC + slag have confirmed that PACl changes the structure of C-S-H gel to C-(A)-S-H gel by supplying aluminum ions [16,26,27].

PACl contains both aluminum and chloride. Therefore, an increase in the PACl content increases the concentration of chloride and aluminum ions in the mixture. Therefore, reactive alumina (Al_2_O_3_^r-^) of pozzolan and tricalcium aluminate (C_3_A) of OPC provide the source of aluminum ions needed to form Friedel’s salts in OPC [28,29]. The supply of aluminum ion has already been reported to play an important role in the formation of Friedel’s salt. Increasing the concentration of PACl increases not only chloride but also aluminum concentration. This eventually influences the formation of Friedel’s salt [15,23,30]. Finally, C-S-H gel adsorbs more chloride ions. Consequently, the peaks for Friedel’s salt gradually increase as the PACl content increases, as Figure 1c shows.

### 3.2. Pore Structure

**Figure 2:**
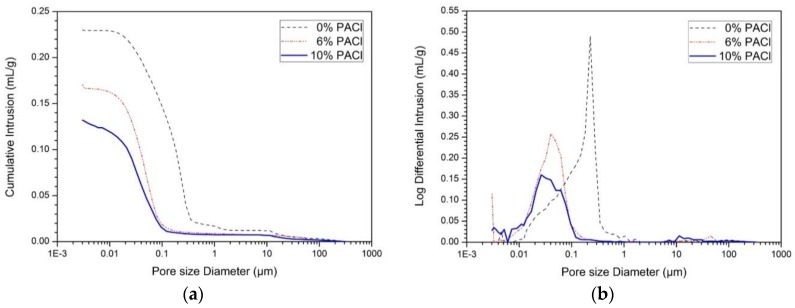
Results of mercury intrusion porosimetry, which gives (**a**) the cumulative intrusion and (**b**) the log differential intrusion as a function of pore diameter.

Figure 2 shows the cumulative intrusion as a function of pore diameter for the 28 day sample. In Figure 2a, the pore volume and size decrease as the PACl content increases from 0% to 6%, and to 10%. Figure 2b shows that as the amount of PACl increases to 0%, 6%, and 10%, the pore diameters decrease to 0.27, 0.041, and 0.0273 µm, while the graph shifts to the left as a whole and the peak height decreases. 

According to Mindess et al. [31], the pores in cement paste may be divided into large-capillary pores (10–0.05 µm), medium-capillary pores (0.05–0.01 µm) and gel pores (<0.01 µm). The gel pores give the intrinsic porosity of C-S-H. Table 3 shows the ratio of total porosity and pore size from 0%, 6%, and 10% PACl samples from MIP results. As the PACl content increases, the concentration of large-capillary pores decreases and that of medium capillary and gel pores increases. In particular, the highest gel-pore percent is measured to be 15.2% for 10% PACl. The increased gel porosity may be due to the increased amount of C-(A)-S-H in the paste. As the PACl contents increased to 0%, 6%, and 10%, the total porosity decreased to 41.9%, 30.9%, and 23.0%. Therefore, an increase in PACl content reduces the pore size and the amount of reaction product, resulting in a compact matrix.

The reduced pore size and volume upon PACl addition is due to two actions: The first is the structural change of C-S-H gel due to the incorporation of aluminum ions from PACl [24]. Increasing the PACl content increases the aluminum-ion concentration in the mixture, which promotes the transformation of C-S-H gel into the denser C-(A)-S-H gel [16,32,33]. In addition, the aluminum ion of PACl improves the polymerization of C-S-H gel to form the denser C-(A)-S-H gel reaction product [34], which increases gel porosity.

The second action is the formation of Friedel’s salt from the chloride ions of PACl. Friedel’s salts are mainly formed in the pores, thereby filling the pores in the matrix [29,35] and reducing the overall pore volume. Thus, an increased PACl content also increases the concentration Friedel’s salts, as confirmed by the XRD results shown in Figure 1.

### 3.3. Compressive Strength

**Figure 3:**
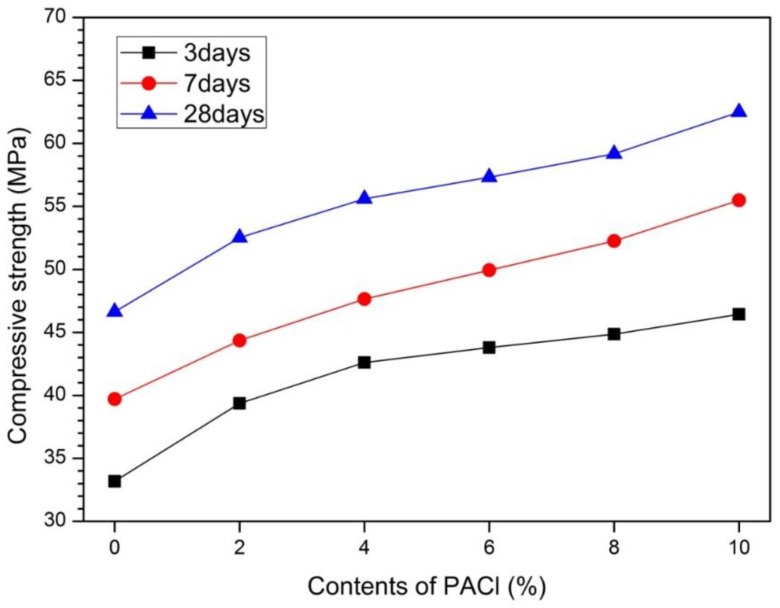
Compressive strength of PACl-added OPC as function of PACl content.

Figure 3 shows the results of measurements of compressive strength of 3, 7, and 28 day samples as a function of PACl content. As shown in Figure 3, the compressive strength increases at all sample ages as the PACl content increases from 0% to 10%. The 3 day compressive strength of 0% PACl is 33.2 MPa. As the PACl content increases, the compressive strength gradually increases to 46.4 MPa at 10% PACl. Thus, the compressive strength of PACl-added OPC increases continuously from 3 to 28 days.

To confirm this increase in compressive strength, we measured the compressive strength as a function of PACl content at each sample age. The increase in compressive strength when adding PAC1 is due to the high reactivity of liquid PAC1 in the initial hydration stage and promotes the formation of reactants such as Friedel’s salt and C-(A)-S-H gel. In addition, the greatest increase in compressive strength occurs for 10% PACl content, independent of sample age. For example, with respect to 0% PACl, the increase in strength for 10% PACl is 139.9% for 3 day samples, 139.7% for 7 day samples, and 133.9% for 28 day samples.

The increase in PACl content increases the aluminum-ion concentration, which in turn increases the content of dense C-(A)-S-H gel. The increase in C-(A)-S-H gel increases the strength by rendering the matrix more compact [32,33,36,37]. In particular, the increase in gel-pore diameter (<0.01 μm) in Figure 2 means that the production of dense C-(A)-S-H gel increases. Friedel’s salts formed from chloride ions from PACl also fill the pores in the reaction product matrix, increasing the compressive strength further [29,35]. Thus, the reduction of pores due to filling by Friedel’s salts (already mentioned in conjunction with Figure 1) and the MIP results in Figure 2 show that the matrix densifies and becomes stronger.

### 3.4. Microstructure

**Figure 4:**
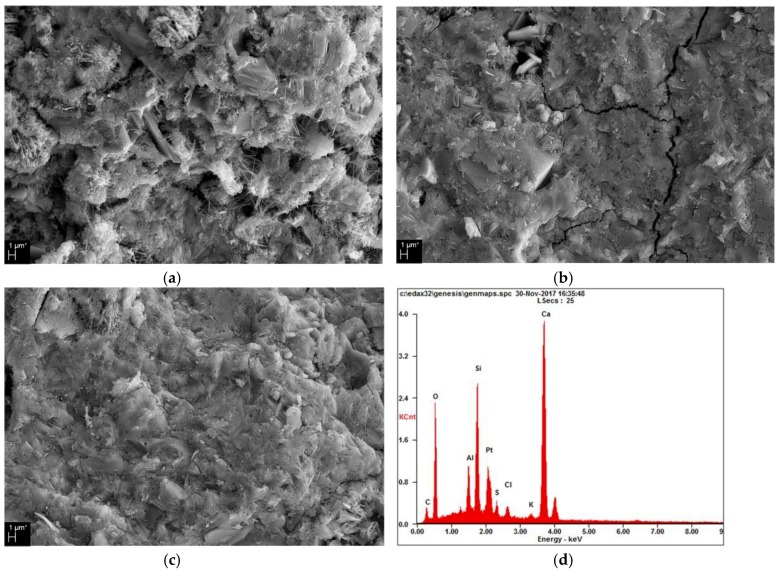
Scanning electron microscopy (SEM) images of (**a**) 0% PACl, (**b**) 6% PACl, and (**c**) 10% PACl. (**d**) Results of energy-dispersive X-ray spectroscopy (EDS) analysis of 10% PACl.

Figure 4a–c show SEM images of 0%, 6%, and 10% PACl samples, respectively. The 0% PACl sample has a rough fracture surface and many pores. Long needle-like ettringite also appears inside and near the pores. This supports the XRD results shown in Figure 1 regarding the ettringite peak.

The 6% PACl sample is compact and less rough than the fracture surface of the 0% PACl sample. In addition, a small number of pores appears and the hydration products form a dense matrix. The 10% PACl sample shows that the pores are reduced further and the matrices of the 6% and 10% PACl samples shown in Figure 4b,c support the aforementioned increase in compressive strength. 

As already mentioned in conjunction with Figure 3, the compressive strength increases as the PACl content increases for all the sample ages. Figure 4d shows the energy-dispersive X-ray spectroscopy (EDS) analysis of the reactants at arbitrary points within the SEM image for the 10% PACl sample shown in Figure 4c. The peaks corresponding to Ca-Al-Si and C of Friedel’s salts are apparent. The SEM image shows the C-(A)-S-H gel and Friedel’s salts that are detected in the XRD results of Figure 1.

**Figure 5:**
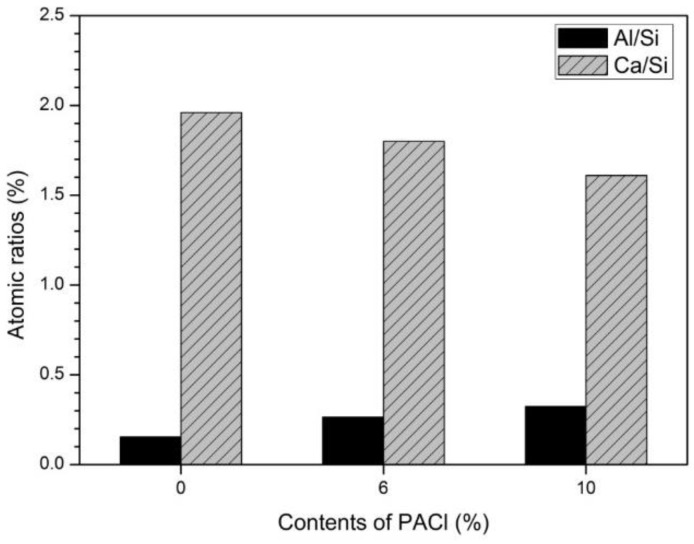
Atomic ratios of hydration products for 0%, 6%, and 10% PACl samples.

Figure 5 shows the average atomic ratio obtained from EDS analysis at 15 arbitrary points of the reactants shown in Figure 4. The Ca/Si ratio of the C-S-H gel, which is a general OPC hydration product, is about 1.5–1.9 [25,38]. With increasing PACl content, the Ca/Si ratio decreases and the Al/Si ratio increases.

The decreasing Ca/Si ratio with increasing PACl content is due to accelerated hydration of cement particles by PACl, which is consistent with the results of a previous study that blended OPC-slag and PACl [16]. The increasing Al/Si ratio with increasing PACl content is also influenced by the aluminum ion contained in PACl. C-S-H gel is a typical OPC hydration product and affects the mechanical and chemical properties of OPC. Previous studies have shown that the Ca/Si and Al/Si ratios of C-S-H gel depend on various factors [39,40,41]. In addition, C-S-H gel absorbs various ions such as aluminum, sodium, and potassium, which modifies the gel structure [42,43]. When C-S-H gel absorbs aluminum ions, its structure changes to that of C-(A)-S-H gel. Previous studies have shown that these structural changes are due to the concentration of mixed aluminum [44,45], the Ca/Si ratio of C-S-H gel [24], and the pH of the solution [24,25].

To clarify the structure and factors that influence C-S-H gel and C-(A)-S-H, several groups have studied the structure of C-(A)-S-H gels as a function of the Ca/Si and Al/Si ratios [37,41,46,47]. The increase in the Al/Si ratio is due to the increase in aluminum absorbed by the C-S-H gel as the aluminum concentration increases [24,25]. Previous studies have shown that the Al/Si ratio of C-S-H gel increases with aluminum concentration when Ca/Si ≥ 1.0 [48,49,50]. In the present study, the Ca/Si ratio for 0% PACl is 1.96 and the Al/Si ratio increases with increasing PACl content.

The reason that the Ca/Si ratio decreases with increasing PACl content may be deduced as follows: The decrease in the Ca/Si ratio of C-S-H gel is caused by the increase in silica chain length in the C-S-H gel [25], which exerts a strong effect on the aluminum ion of PACl. Specifically, aluminum is shifted to the neighboring bridging position of the dreierketten chain of the C-S-H gel, allowing the silica chain to lengthen [25]. As the silica chain of C-S-H gel lengthens, the silicon concentration increases and the calcium concentration decreases, so the Ca/Si decreases [26,51]. The increased aluminum concentration that occurs with increasing PACl content increases the silicon concentration and decreases the calcium and hydroxide concentrations [25], again contributing to a decrease in the Ca/Si ratio.

As the aluminum-ion concentration increases in the mixture, aluminum is absorbed by the C-S-H gel and some is used to form katoite or stratlingite [24]. However, the XRD results of Figure 1b show no evidence of katoite or stratlingite, even though the PACl content increased. The reason for this result is related to the Al/Si ratio. L’Hôpital et al. [24] reported that the aluminum concentration decreases with the formation of katoite, stratlingite, or Al(OH)_3_, the latter of which is an aluminum-ion-containing material with Al/Si = 0.33. In the present study, the increase in PACl content increases the aluminum-ion concentration, therefore, more aluminum ions are absorbed by the C-S-H gel, which leads primarily to the formation of C-(A)-S-H gel. Consequently, the Al/Si ratio of the reactant (C-(A)-S-H gel) increases, although it remains too low to form katoite or stratlingite. In Figure 5, the Al/Si ratio of 10% PACl is 0.32, which is too low to form katoite or stratlingite. Therefore, no peaks for katoite or stratlingite appear in the XRD spectra shown in Figure 1.

**Figure 6:**
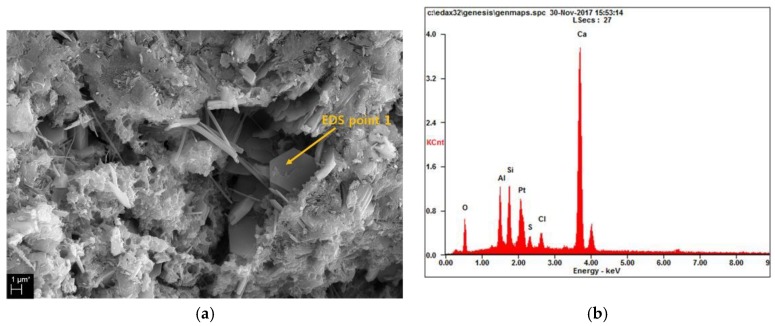

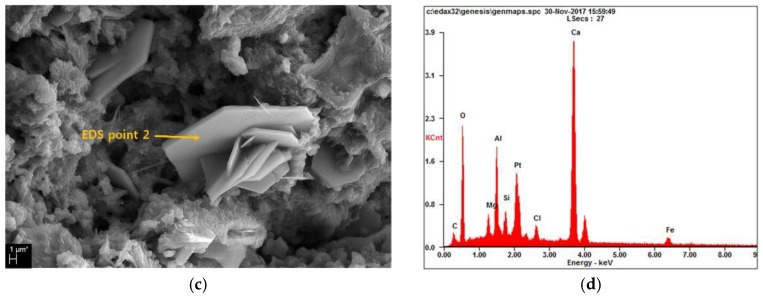
SEM images (**a**) Friedel’s salt (hexagonal plates) with 6% PACl. (**b**) Results of energy dispersive spectroscopy (EDS) analysis at “EDS point 1” shown in panel (**a**). (**c**) Friedel’s salt with 10% PACl. (**d**) Results EDS analysis at “EDS point 2” shown in panel (**c**).

Figure 6 shows the hexagonal plates of Friedel’s salt found in 6% and 10% PACl samples [52]. Friedel’s salt appears mainly in the pores of the matrix. In Figure 6a of 6% PACl, needle-like ettringite appears together with Friedel’s salts. Figure 6c shows Friedel’s salts in a 10% PACl sample. The hexagonal plates are similar in shape to the 6% PACl sample. Figure 6b,d show the results of EDS analysis of Friedel’s salts in 6% and 10% PACl samples, respectively. The presence of Friedel’s salts as revealed by SEM images and EDS analysis supports the XRD results of Figure 1, which also indicate the presence of Friedel’s salts. Thus, the contribution of Friedel’s salts to the reduced size and number of pores indicated by the MIP results of Figure 2 can be explained essentially by observing the inside of the pores.

### 3.5. Thermal Analysis

**Figure 7:**
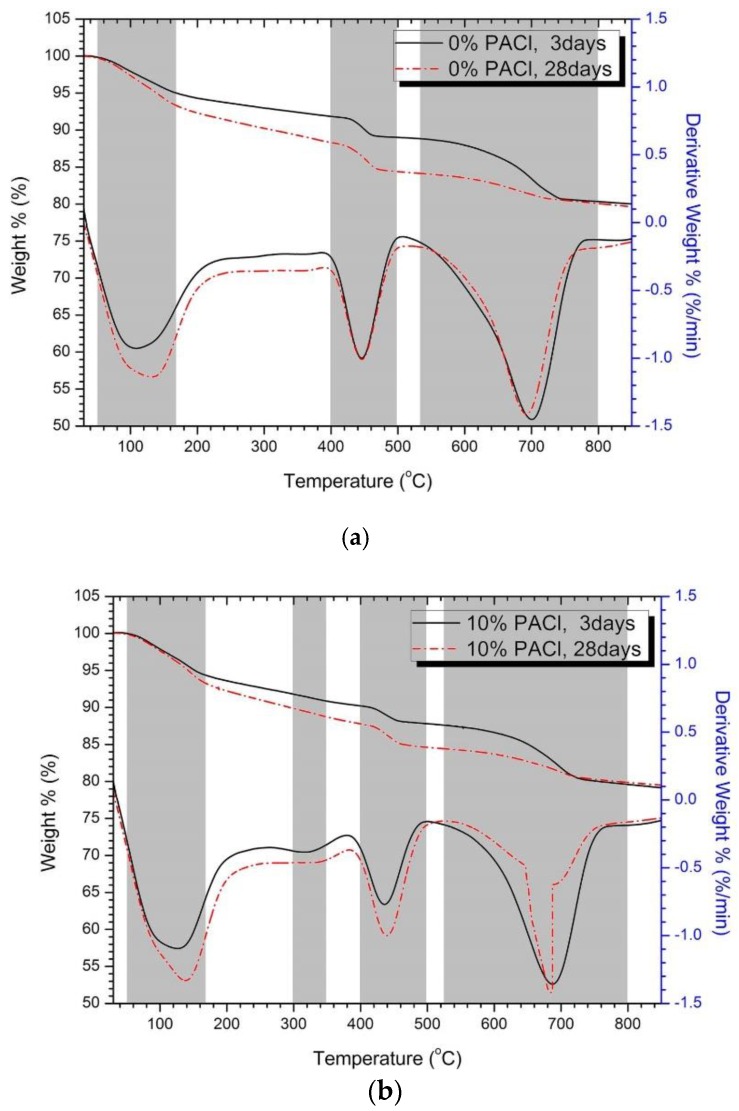
Results of thermogravimetric and differential thermogravimetric analysis: (**a**) 0% PACl, (**b**) 10% PACl.

Samples of varying PACl content were subjected to a thermal analysis of the reactants. Figure 7 shows the results of a thermogravimetric and differential thermogravimetric analysis applied to 3 day and 28 day samples with 0% and 10% PACl. In the thermal analysis graphs of 0% and 10% PACl, the weight-loss temperature ranges are 50‒140, 400‒500, and 530‒800 °C (see gray bands in Figure 7a,b). Each weight-loss band indicates the loss of water from the reactant.

The first weight loss between 50–180 °C observed for all samples was due to the decomposition of C-S-H or C-(A)-S-H gel [53,54,55]. At the same time, it was attributed to the release of evaporable water and the start of the dehydration of ettringite [54,55,56,57]. The weight loss at 28 days is greater than that at 3 days for both 0% PACl (Figure 7a) and 10% PACl (Figure 7b), which means that hydration products continue to form up to 28 days, thereby increasing the amount of hydration products. In the XRD spectra shown in Figure 1, the ettringite and C-(A)-S-H peaks at 28 days are smaller than at 3 days. However, the thermal analysis indicates that the hydration products increase with increasing age. Note also that the weight loss of 10% PACl exceeds that of 0% PACl. The increase in PACl content increases the aluminum-ion concentration in the mixture, which promotes the formation of C-(A)-S-H gel [16,32,33]. Therefore, adding PACl accelerates the hydration reaction of cement particles and increases the amount of reaction products.

Secondly, weight loss in the 400–500 °C temperature range was due to the dehydroxylation of portlandite [53,54,58]. As Figure 7a shows, the weight loss of portlandite in 0% PACl is the same after 3 days as after 28 days. However, as Figure 7b shows, the weight loss of 10% PACl is greater after 28 days than after 3 days, which is because the hydration of cement is promoted with increasing age and the production of portlandite increases. The hydration-promoting action of PACl was already mentioned in a previous study in which powder-type PACl was mixed with OPC-GGBFS binder [16]. Similar results are obtained in the present study by using liquid PACl.

The third weight loss band observed in the 530–800 °C temperature range was likely due to the decomposition of calcite [53,58]. The calcite weight loss for 0% PACl after 3 days and after 28 days is almost identical. However, the calcite weight loss for 10% PACl after 28 days is less than that after 3 days, which is likely due to the fact that most of the calcium eluted by the hydration of the cement is used to form ettringite or C-(A)-S-H gel, so less calcite is formed. This result is consistent with the XRD results for 10% PACl (see Figure 1b).

Figure 7b shows a small weight-loss band for 10% PACl at 300–350 °C, which does not appear for 0% PACl. This weight loss is due to Friedel’s salts [15,53]. As already mentioned in conjunction with Figure 1, the XRD peak for Friedel’s salts increases as the PACl content increases. Thermal analysis shows that the increase in PACl content promotes the formation of C-(A)-S-H gel and confirms the formation of the new reaction product (i.e., Friedel’s salts). These reaction products are created by the aluminum and chloride ions in PACl. Thus, PACl promotes the hydration of cement and the formation of hydration products.

## 4. Conclusions

The conclusions from the experimental results on the characteristics of OPC mixed with PACl are summarized as follows.

PACl promotes the formation of Friedel’s salts and reduces portlandite. This change in hydration reactant is evident as the contents of PACl increases. The chloride and aluminum ions contained in PACl, together with the calcium supplied from OPC, influence the formation of Friedel’s salt. Friedel’s salts were observed by XRD and thermal analysis, and SEM observations showed hexagonal plate formation. Some aluminum contributes to the formation of C-(A)-S-H gel. This can be inferred from thermal analysis and supported by the analysis of Al/Si and Ca/Si ratios in the EDS analysis of hydration reactants. Therefore, it was confirmed that chloride ions and aluminum ions of PACl had a great influence on the hydration reactant.

PACl also caused a change in pore structure. As the amount of PACl increased, the total porosity decreased from 41.9% of 0% PACl to 23.0% of 10% PACl. PACl also reduces large-capillary pores (10–0.05 µm) and increases medium-capillary pores (0.05–0.01 µm) and gel-pores (<0.01 µm). The result is an effect of reducing the diameter of the pores. The decrease in pore diameter is due to the formation of a dense matrix due to the formation of C-(A)-S-H gel and the pore filling effect by Friedel’s salt. This change in pore structure affected the improvement of compressive strength. PACl improved compressive strength by changing hydration reactant and pore structure. The compressive strength improvement was observed in all measurement ages at 3, 7, and 28 days, with 10% PACl showing the highest compressive strength value. The compressive strength increases of 10% PACl reached 139.9% at 3 days, 139.7% at 7 days, and 133.9% at 28 days.

It was found that PACl has an effect of improving the mechanical performance by causing the change of hydration reactant and pore structure of OPC paste. However, chloride ions contained in PACl are a risk factor for corrosion of rebar or steel. Therefore, further studies are needed to examine the corrosion effects of rebar in PACl-containing concrete. Or, you can examine the applicability to bricks and panels that do not use rebar, and concrete members that use fiber reinforced plastic (FRP)-bar instead of rebar.

## Figures and Tables

**Table 1:** Properties of ordinary Portland cement (OPC).

	**Chemical Components (%)**							**Density** **(g/mm^3^)**	**Fineness** **(m^2^/kg)**	**LOI** **(%)**
OPC	SiO_2_	Al_2_O	Fe_2_O	MgO	CaO	K_2_O	SO_3_			
	21.75	4.83	3.52	4.04	62.16	0.67	2.83	0.00315	330	0.74

**Table 2:** Properties of liquid-type polyaluminum chloride (PACl) (provided by the manufacturer).

**Chemical Components (%)**				**Density** **(g/mm^3^)**	**pH**	**Basicity** **(%)**
Al_2_O	Fe_2_O	SO_3_	Cl^-^			
17.27	0.01	0.30	21.33	0.00137	4.01	40

**Table 3:** Total porosity and pore size analysis.

Level	0% PACl	6% PACl	10% PACl
Micro pores (>10 µm; %)	1.1	2.7	4.0
Large-capillary pores (10–0.05 µm; %)	81.7	35.6	28.2
Medium-capillary pores (0.05–0.01 µm; %)	16.1	52.3	52.6
Gel pores (<0.01 µm; %)	1.1	9.4	15.2
Total porosity (%)	41.9	30.9	23.0
